# Oxidative Status before and after Renal Replacement Therapy: Differences between Conventional High Flux Hemodialysis and on-Line Hemodiafiltration

**DOI:** 10.3390/nu11112809

**Published:** 2019-11-17

**Authors:** José Alberto Navarro-García, Elena Rodríguez-Sánchez, Jennifer Aceves-Ripoll, Judith Abarca-Zabalía, Andrea Susmozas-Sánchez, Laura González Lafuente, Teresa Bada-Bosch, Eduardo Hernández, Evangelina Mérida-Herrero, Manuel Praga, Luis Miguel Ruilope, Gema Ruiz-Hurtado

**Affiliations:** 1 Cardiorenal Translational Laboratory, Institute of Research i+12, Hospital Universitario 12 de Octubre, 28021 Madrid, Spain; jalbertong@gmail.com (J.A.N.-G.); elena.rodsanchez@gmail.com (E.R.-S.); jen.ace.rip@hotmail.com (J.A.-R.); judithrit@hotmail.com (J.A.-Z.); andreasusmozas@gmail.com (A.S.-S.); laura.gonzlafuente@gmail.com (L.G.L.); ruilope@icloud.com (L.M.R.); 2 Service of Nephrology, Hospital Universitario 12 de Octubre, 28021 Madrid, Spain; teresa_bada@hotmail.com (T.B.-B.); ehm3871@yahoo.es (E.H.); evameridaherrero@hotmail.com (E.M.-H.); mpragat@senefro.org (M.P.); 3 School of Doctoral Studies and Research, European University of Madrid, 28670 Madrid, Spain; 4 CIBER-CV, Hospital Universitario 12 de Octubre, 28021 Madrid, Spain

**Keywords:** oxidative stress, dialysis, on-line hemodiafiltration, high-flux dialysis

## Abstract

Hemodialysis patients experience high oxidative stress because of systemic inflammation and depletion of antioxidants. Little is known about the global oxidative status during dialysis or whether it is linked to the type of dialysis. We investigated the oxidative status before (pre-) and after (post-) one dialysis session in patients subjected to high-flux dialysis (HFD) or on-line hemodiafiltration (OL-HDF). We analyzed carbonyls, oxidized LDL (oxLDL), 8-hydroxy-2′-deoxyguanosine, and xanthine oxidase (XOD) activity as oxidative markers, and total antioxidant capacity (TAC), catalase, and superoxide dismutase activities as measures of antioxidant defense. Indices of oxidative damage (OxyScore) and antioxidant defense (AntioxyScore) were computed and combined into a global DialysisOxyScore. Both dialysis modalities cleared all markers (*p* < 0.01) except carbonyls, which were unchanged, and oxLDL, which increased post-dialysis (*p* < 0.01). OxyScore increased post-dialysis (*p* < 0.001), whereas AntioxyScore decreased (*p* < 0.001). XOD and catalase activities decreased post-dialysis after OL-HDF (*p* < 0.01), and catalase activity was higher after OL-HDF than after HFD (*p* < 0.05). TAC decreased in both dialysis modalities (*p* < 0.01), but remained higher in OL-HDF than in HFD post-dialysis (*p* < 0.05), resulting in a lower overall DialysisOxyScore (*p* < 0.05). Thus, patients on OL-HDF maintain higher levels of antioxidant defense, which might balance the elevated oxidative stress during dialysis, although further longitudinal studies are needed.

## 1. Introduction

Chronic kidney disease (CKD) is characterized by the progressive loss of renal function [1]. The best overall indicator of kidney function is the glomerular filtration rate (GFR), which is defined as the volume of plasma that can be completely cleared of a particular impurity in a unit of time. CKD progresses as GFR decreases, reaching an advanced stage (stage 5) at GFR values lower than 15 mL/min/1.73 m^2^, which is considered end-stage renal disease (ESRD) and necessitates renal replacement therapy (RRT)—dialysis or renal transplantation—quickly [2]. Dialysis is the most common form of RRT and consists of the filtration of the patient’s blood using a dialyzer, which is based on two main physical processes: diffusion and convection. There are different types of dialysis according to the diffusion and convection grades, of which hemodialysis is the most widely used. Hemodialysis provides a temporary solution for renal dysfunction, as it replaces some filtration functions of the kidney, but morbidity and mortality remain high because of inflammation and its complications, such as cardiovascular disease or associated oxidative stress [3]. Indeed, hemodialysis courses with inflammation in 30–50% of patients [3,4]. Another form of dialysis with higher convection volumes is on-line hemodiafiltration (OL-HDF), which offers a superior clearance of uremic substances with a greater spectrum of molecular sizes. Importantly, this modality has been shown to reduce mortality when compared with hemodialysis if high convection volumes are reached [5]; however, the mechanisms involved in this improvement are not known.

Oxidative stress is a pathological state in which the production of reactive oxygen species (ROS) exceeds the scavenging capacity of antioxidant systems. ROS are mainly produced by mitochondria, as by-products of respiration, and by pro-oxidant enzymes such as xanthine oxidase (XOD). The majority of ROS are scavenged by antioxidant systems, including the enzymes superoxide dismutase (SOD) and catalase, and low-molecular weight antioxidant molecules including bilirubin, glutathione, and vitamins. ROS levels increase in states of oxidative stress, and can induce reversible or irreversible damage to proteins, lipids, and DNA, which has direct consequences for the physiological function of cells and tissues [6]. These oxidative modifications to macromolecules are the preferred surrogate markers for determining oxidative damage, as ROS are very difficult to measure due to their extremely short half-life. A common limitation in the analysis of oxidative stress is that most studies survey only a few markers of oxidative damage, and neglect to consider the physiological antioxidant capacity. Because oxidative stress is a multifactorial state that encompasses multiple mechanisms of both oxidative damage and antioxidant defense [7], it would seem prudent to approach its analysis by considering multiple biomarkers, and combining them into a “multimarker” score indicating the global oxidative status.

The progression from CKD to ESRD is associated with an increase in oxidative stress because the kidney is one of the main sources of antioxidant systems, and because the accumulation of uremic toxins further triggers the build-up of ROS [8], leading to a vicious cycle of oxidative stress and inflammation [9,10,11,12]. Of note, oxidative stress is more pronounced in patients undergoing dialysis, not only because of the decline in renal function, but also because of the treatment itself. For example, (i) the dialysis membrane and dialysate activate leukocytes, which in turn increase inflammation and ROS production; (ii) antioxidant systems, especially those with low or very-low molecular weight, are filtered during dialysis; and (iii) patients on dialysis have strong restrictions on fruit and vegetable intake, which reduces their intrinsic antioxidant capacity because of the low ingestion of vitamins and polyphenols [13]. In this regard, there have been differences observed in the oxidative state in patients under hemodialysis depending on the type of membrane used [14], and between hemodialysis and peritoneal dialysis treatments [15,16]. Yet, there is a lack of knowledge about the possible differences in the oxidative status of patients treated with hemodialysis versus OL-HDF, and before and after dialysis.

The aim of the present study was to assess and compare the global oxidative status before and after hemodialysis using high-flux membranes (high-flux dialysis (HFD)) and OL-HDF, and using a multimarker approach that considers both pro-oxidant modifications and antioxidant systems.

## 2. Materials and Methods

### 2.1. Study Population

**Figure 1:**
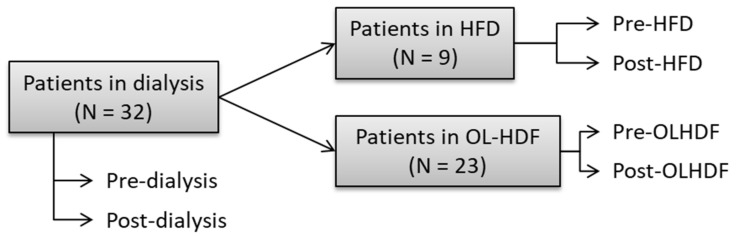
Schematic flow-chart of patient selection and sample recruitment.

This cross-sectional study included 32 dialysis-dependent patients with CKD recruited to the dialysis service of the Hospital Universitario 12 de Octubre (Madrid, Spain). Of these subjects, 9 patients were treated with conventional HFD and 23 were treated with OL-HDF. Figure 1 shows the flow-chart of patient selection and sample recruitment. Causes of renal failure were as follows: chronic glomerulonephritis (21.9%), diabetic nephropathy (15.6%), polycystic kidney disease (12.5%), interstitial nephritis (9.4%), atypical hemolytic uremic syndrome (6.3%), malignant hypertension (6.3%), nephroangiosclerosis (6.3%), ischemic nephropathy (3.1%), and others (18.8%). Subjects underwent clinical examinations before dialysis and blood analysis before (pre-) and after (post-) one dialysis session. Blood samples were collected in EDTA or heparin tubes and immediately centrifuged at 2000 rpm for 10 min. Plasma samples were stored at −80 °C until use. All patients signed an informed consent. The study was approved by the local ethics committee of Hospital Universitario 12 de Octubre (CEI: 16/250) in compliance with the recommendations of the Declaration of Helsinki.

### 2.2. Assessment of Oxidative Damage and Antioxidant Defense

#### 2.2.1. Oxidative Biomarkers

Oxidative modifications in proteins in the form of carbonyl groups were assessed using a 2,4-dinitrophenylhydrazine assay adapted for a microplate reader [17] and expressed as nmol/mL. Oxidized low-density lipoprotein (oxLDL) was measured as a marker of lipid peroxidation using a commercial sandwich ELISA based on the monoclonal antibody 4E6 (Mercodia AB, Uppsala, Sweden). Oxidative damage to DNA was measured by quantification of the nucleoside 8-hydroxy-2′-deoxyguanosine (8-OHdG) using a commercial ELISA kit (StressMarq Biosciences Inc., Victoria, BC, Canada). XOD activity was estimated with the Amplex Red assay (Invitrogen, Carlsbad, CA, USA) and expressed as µU/mL.

#### 2.2.2. Antioxidant Biomarkers

Enzymatic antioxidant defense was determined by measuring catalase and SOD activities. Catalase activity was measured with an Amplex Red assay (Invitrogen) and expressed as U/mL. SOD activity was measured with a colorimetric kit (Invitrogen) and expressed as mU/mL. Plasma total antioxidant capacity (TAC) was measured with a TAC assay [18], based on enhanced horseradish peroxidase-catalyzed luminol chemiluminescence adapted for a microplate reader. Time-dependent luminescence inhibition with respect to hydrogen peroxide was used to calculate the area under the curve (AUC).

All parameters were corrected post-dialysis according to the weight loss during the process of dialysis with the following equation:

(1) $$Cc=\;\frac{Cpost}{1+\frac{BWpre-BWpost}{0.2*BWpost}}$$

where *Cc* is the corrected concentration post-dialysis, *Cpost* is the concentration post-dialysis, *BWpre* is the body weight pre-dialysis, and *BWpost* is the body weight post-dialysis [19].

### 2.3. Calculation of OxyScore, AntioxyScore, and DialysisOxyScore

The biomarkers of oxidative damage and antioxidant defense were combined in a multimarker score of oxidative damage (OxyScore) and antioxidant defense (AntioxyScore), respectively, as described [20,21]. Protein carbonyls, oxLDL, 8-OHdG, and XOD activity were standardized using the pre-dialysis group as a reference for the OxyScore. Catalase activity, SOD activity, and TAC were equally standardized for the AntioxyScore. Finally, the global index of oxidative status was referred to as the DialysisOxyScore and was calculated by subtracting the AntioxyScore from the OxyScore.

### 2.4. Statistical Analysis

Normality was determined with the Kolmogorov–Smirnov test. Pre- and post-dialysis groups were compared using paired Student’s *t*-test or the Wilcoxon signed-rank test, and HFD and OL-HDF groups were compared using unpaired Student’s *t*-test or the Mann–Whitney U test. Categorical variables were compared with Fisher’s exact test. Results are expressed as mean ± SEM unless otherwise stated, and *p* values < 0.05 were considered significant. Analyses were performed using GraphPad Prism 6 (GraphPad Software Inc., San Diego, CA, USA), and SPSS Statistics v22 (IBM, Armonk, NY, USA).

## 3. Results

### 3.1. Clinical Characteristics

Patients’ baseline characteristics are shown in Table 1. Patients treated with OL-HDF were predominantly male (65.2%), while in the HFD group there was a higher percentage of females (88.9%). Also, body-mass index (BMI) was higher in the OL-HDF group than in the HFD group, and the time in dialysis was longer in those patients treated with OL-HDF with respect to HFD. There were no differences between groups in blood pressure, medical history pathologies, treatments, or N-terminal-pro hormone B-type natriuretic peptide (NT-proBNP), 25-hydroxyvitamin D, total cholesterol or triglycerides, serum creatinine and albumin, CaP, Kt/V, potassium, and bicarbonate.

### 3.2. Dialysis Treatment Increases Oxidative Stress

**Figure 2:**
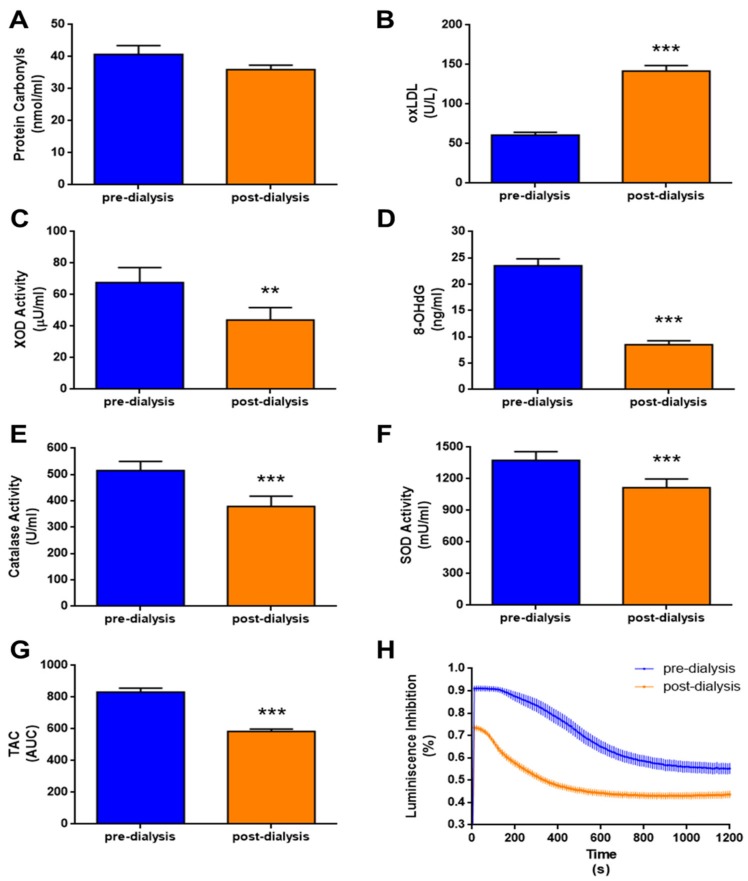
Markers of oxidative damage (**A**–**D**) and antioxidant defense (**E**–**H**) in dialysis patients pre- and post-dialysis. (**A**) Protein carbonyls, (**B**) oxidized LDL (oxLDL), (**C**) xanthine oxidase (XOD) activity, (**D**) 8-hydroxy-2′-deoxyguanosine (8-OHdG), (**E**) catalase activity, (**F**) superoxide (SOD) activity, (**G**) total antioxidant capacity (TAC) measured as AUC, and (**H**) TAC variation in luminescence inhibition after plasma addition (time = 1 s). Data is presented as mean ± SEM. ** *p* < 0.01 and *** *p* < 0.001 vs. pre-dialysis.

Protein carbonyls, oxLDL, XOD activity, and 8-OHdG were measured as oxidative damage biomarkers at pre- and post-dialysis. Globally, no changes were observed post-dialysis in protein carbonyls, as a marker of oxidative protein damage (*p* = 0.168, Figure 2A). However, oxidative damage on lipids, measured as oxLDL, was significantly increased post-dialysis (*p* < 0.001, Figure 2B). By contrast, XOD activity and 8-OHdG levels were significantly lower post-dialysis than pre-dialysis (*p* < 0.01 and *p* < 0.001, respectively, Figure 2C,D). We measured catalase and SOD activities and TAC as biomarkers of antioxidant defense, and all were significantly decreased post-dialysis (*p* < 0.001, Figure 2E–H). The time course of luminescence inhibition during the TAC assay, which was used to calculate AUC values, is represented in Figure 2H.

**Figure 3:**
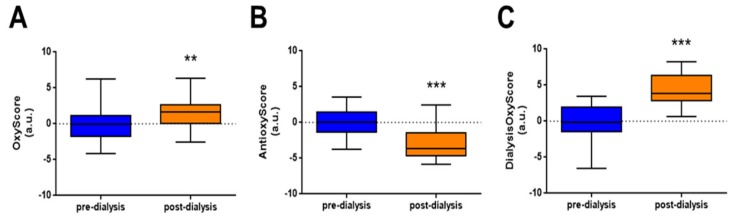
OxyScore, AntioxyScore, and DialysisOxyScore in pre- and post-dialysis stages. (**A**) OxyScore, (**B**) AntioxyScore, and (**C**) DialysisOxyScore in dialysis patients. Data is presented as median ± interquartile range. ** *p* < 0.01 and *** *p* < 0.001 vs. pre-dialysis.

The multimarker score of oxidative damage, OxyScore, was calculated as the sum of the XOD pro-oxidant activity and oxidative damage in proteins (carbonyls), lipids (oxLDL), and DNA (8-OHdG). The OxyScore increased globally post-dialysis (*p* < 0.05, Figure 3A). The AntioxyScore, as a multimarker score of antioxidant defense, was computed as the sum of TAC and the enzymatic antioxidant activities of SOD and CAT. In contrast to the OxyScore, patients presented a significant decrease in the AntioxyScore post-dialysis (*p* < 0.001, Figure 3B). The global oxidative status dialysis score, DialysisOxyScore, was calculated as the difference between OxyScore and AntioxyScore, and was increased after dialysis treatment (*p* < 0.001, Figure 3C).

### 3.3. Oxidative Stress and Antioxidant Defense Markers Are Different between HFD and OL-HDF Modalities

**Figure 4:**
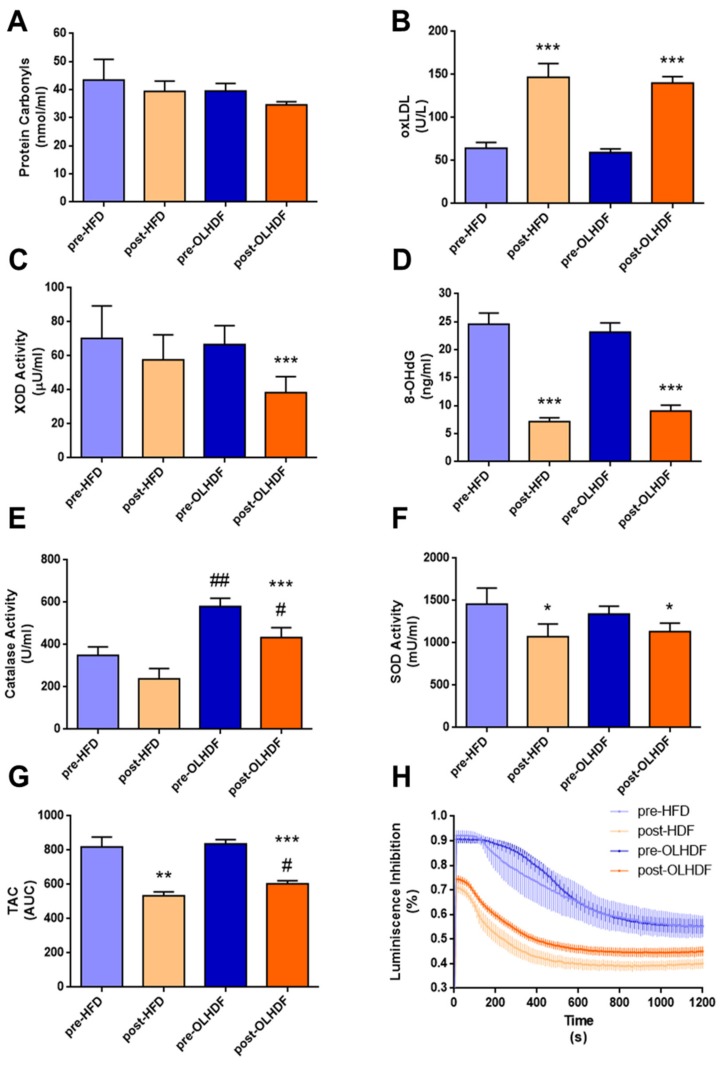
Markers of oxidative damage (**A**–**D**) and antioxidant defense (**E**–**H**) in HFD and OL-HDF dialysis patients pre- and post-dialysis. (**A**) Protein carbonyls, (**B**) oxidized LDL (oxLDL), (**C**) xanthine oxidase (XOD) activity, (**D**) 8-hydroxy-2′-deoxyguanosine (8-OHdG), (**E**) catalase activity, (**F**) superoxide (SOD) activity, (**G**) total antioxidant capacity (TAC) measured as AUC, and (**H**) TAC variation in luminescence inhibition after plasma addition (time = 1 s). Data is presented as mean ± SEM. Blue bars represent data of pre-dialysis samples, clear blue for HFD, and dark blue for OL-HDF. Orange bars represent data of post-dialysis samples, clear orange for HDF, and dark orange for OL-HDF. * *p* < 0.05 ** *p* < 0.01 and *** *p* < 0.001 vs. pre-dialysis situation; ^#^ *p* < 0.05 and ^##^ *p* < 0.01 vs. HFD.

We next compared the two dialysis modalities with respect to changes in oxidative stress and antioxidant defense. No changes were observed in protein carbonyls post-dialysis independently of the type of dialysis (Figure 4A). However, oxLDL significantly increased in both treatment groups post-dialysis (*p* < 0.001, Figure 4B). By contrast, XOD activity decreased post-dialysis, but only in those patients treated with OL-HDF (*p* < 0.001, Figure 4C). Both modalities significantly reduced 8-OHdG levels (*p* < 0.001, Figure 4D). With respect to antioxidant defense, a different pattern was observed when we compared the biomarkers of antioxidant defense between HFD and OL-HDF treatments. Patients treated with OL-HDF presented with significantly higher catalase activity pre-dialysis when compared with HFD-treated patients (*p* < 0.01, Figure 4E). No differences were observed between groups in SOD activity or TAC (Figure 4F,G) in pre-dialysis samples. When the effect of dialysis was analyzed in both patient groups, we observed a significant reduction of catalase activity only in the OL-HDF group post-dialysis (*p* < 0.001, Figure 4E). Nevertheless, catalase activity remained higher in OL-HDF patients than in HFD patient’s post-dialysis (*p* < 0.05, Figure 4E). SOD activity was significantly reduced in both dialysis groups post-dialysis (*p* < 0.05, Figure 4F). Also, both modalities decreased TAC activity post-dialysis (*p* < 0.01 and *p* < 0.001, respectively, Figure 4G), although TAC activity remained higher in OL-HDF patients compared with HFD patient’s post-dialysis (*p* < 0.05, Figure 4G). The time course of luminescence inhibition during the TAC assay, which was used to calculate AUC values, is represented in Figure 4H.

### 3.4. Global Oxidative Status (DialysisOxyScore) is Lower after OL-HDF Treatment

**Figure 5:**
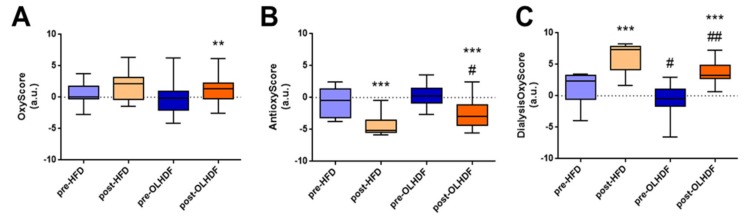
OxyScore, AntioxyScore and DialysisOxyScore in HDF and OL-HDF pre- and post-dialysis. (**A**) OxyScore, (**B**) AntioxyScore, and (**C**) DialysisOxyScore in HFD and OL-HDF patients. Data is presented as median ± interquartile range. Blue bars represent data of pre-dialysis samples, clear blue for HFD, and dark blue for OL-HDF. Orange bars represent data of post-dialysis samples, clear orange for HDF, and dark orange for OL-HDF. ** *p* < 0.01 and *** *p* < 0.001 vs. pre-dialysis; ^#^ *p* < 0.05 and ^##^ *p* < 0.01 vs. HFD.

When we compared the OxyScore in pre-dialysis samples, we found no differences between the HFD and OL-HDF groups. OxyScore increased significantly in the OL-HDF group post-dialysis (*p* < 0.05, Figure 5A), although no significant differences were observed in post-dialysis samples between OL-HDF and HFD groups. In both groups, dialysis treatment decreased the AntioxyScore (*p* < 0.001, Figure 5B) but it remained significantly higher in the OL-HDF group compared with the HFD group post-dialysis (*p* < 0.05, Figure 5B). The global oxidative score, DialysisOxyScore, was significantly lower in pre-dialysis samples from the OL-HDF group than from the HFD group (*p* < 0.05, Figure 5C). Although both types of dialysis increased the DialysisOxyScore (*p* < 0.001, Figure 5C), it remained significantly lower in the OL-HDF group (*p* < 0.01, Figure 5C).

## 4. Discussion

This study shows that there is an increase in oxidative stress, mainly driven by oxLDL, together with a decrease in antioxidant systems post-dialysis. However, the decrease in antioxidant systems was lower in the OL-HDF group than in the HFD group, suggesting that oxidative stress might be alleviated in patients treated with OL-HDF.

During the initiation of the dialysis process, the membrane and dialysate induced inflammation and an important increase in ROS production from activated leukocytes, but this increase was stabilized at the end of dialysis [22,23]. Nevertheless, the increase in ROS production might stimulate long-term oxidative damage in proteins, DNA, and lipids. In the present study, no changes in carbonyls were observed post-dialysis, which agrees with previous reports examining protein carbonyls and advanced oxidation protein products [24,25]. However, protein carbonyls have been found to increase in other studies [22,26], which might be due to the different types of dialysis membrane used [14]. Also, XOD activity and 8-OHdG levels were significantly decreased post-dialysis in the present study, suggesting that both markers are efficiently filtered during the dialysis process. By contrast, levels of oxLDL increased post-dialysis, which again agrees with some studies and reflects its major contribution to oxidative stress in patients undergoing hemodialysis [27,28,29], but contrasts with other studies on hemodialysis that show conflicting results [25,30,31]. However, in most published studies malondialdehyde is measured as a marker of lipid peroxidation. Given that dialysis patients are at high risk for cardiovascular complications and atherosclerosis [32] and that oxLDL as a marker of lipid peroxidation is tightly associated with atherosclerosis [33] and cardiovascular risk [21], we considered that oxLDL would better reflect the oxidative status on lipids of dialysis patients. In this sense, our results support that lipid peroxidation is likely the leading cause of the increase in oxidative stress post-dialysis, and indicates that the high risk of developing atherosclerosis in dialysis might be caused in part by the dialysis treatment itself. Therefore, although dialysis is able to filter-out pro-oxidant enzymes such as XOD and products of oxidation such as 8-OHdG, the large elevation in oxLDL promotes an overall increase in OxyScore post-dialysis.

We found that all measured markers of antioxidant defense decreased post-dialysis. Indeed, previous studies have described a decrease in the abundance of low-molecular weight antioxidants and vitamins [14,28,29,30,31,34,35], which has been measured synergistically in TAC assay in our study. This is probably due to the filtration process, or perhaps because these markers are consumed in a physiological effort to counteract the increase in ROS. In this line, Clermont et al. described a reduction in vitamin C post-dialysis together with an increase in products of vitamin C oxidation [35]. This might also explain the reduction in the plasma activity of catalase and SOD. In contrast to the present analysis, catalase and SOD have been described to increase post-dialysis [14,34]. However, these studies measured enzymatic activity in erythrocytes rather than in plasma, as measured herein. However, it is possible that the expression of antioxidant systems increases during dialysis in an attempt to counteract the increase in oxidative stress [14,28], although the plasma-free enzymes are filtered in the process. Consequently, plasma AntioxyScore is reduced post-dialysis. This homogeneous decrease in plasmatic antioxidant systems together with the increase in oxLDL induces an imbalance in favor of oxidative damage, which increases the global index of plasmatic oxidative stress, DialysisOxyScore.

Oxidative stress is associated with increased mortality in hemodialysis patients, and it is well documented that oxidative stress is significantly increased in dialysis patients as compared with the general population, which is linked to all-cause and cardiovascular mortality in hemodialysis [36,37,38]. Accumulation of oxidative products, loss of antioxidant molecules during hemodialysis, and dietary restrictions of dialysis patients could all exacerbate this status. In accordance with these studies, the present study shows a significant increase in oxidative markers and a reduction in antioxidant defense as a consequence of dialysis treatment, supporting the notion that hemodialysis increases oxidative products. Increased ROS formation [36], fatty acid oxidation end-products such as malondialdehyde [37], and serum albumin oxidation [38], have all been associated with higher mortality in hemodialysis. Furthermore, the marker of antioxidant defense biological antioxidant potential (BAP) is associated with all-cause but not cardiovascular mortality in hemodialysis [39]. Some authors have described that the reduction in antioxidant defense in hemodialysis is associated with increased all-cause and cardiovascular mortality in these patients [40,41,42]. Moreover, low levels of TAC in hemodialysis patients due to low levels of bilirubin [40] have been related to increased mortality. Also, polymorphisms in glutathione S-transferase M1, which are associated with a reduction in glutathione levels, have been described in hemodialysis [41] to favor oxidative damage in DNA and reduces survival [42] from all-cause and cardiovascular mortality over 25 years of follow-up [43].

Several studies have compared HFD and OL-HDF treatments, finding that OL-HDF is beneficial over HFD. For example, compared with HFD, OL-HDF treatment significantly decreases mortality and risk of non-fatal cardiovascular events [44,45]. Also, the higher volume used in OL-HDF is associated with better survival [5], pointing to the important role of the convection volume of hemodialysis. Independently of the type of dialysis used, the mortality risk burden might be also attenuated by reducing oxidative stress, which could be accomplished by the use of other less aggressive types of dialysis or using antioxidant therapies. Nonetheless, little is known about the oxidative status in patients under OL-HDF. Klouche et al. recently described that inflammation is not affected by OL-HDF and that advanced oxidation protein products are reduced in the process [46]. This would lead to an attenuation in oxidative stress in these patients, which could contribute to their improved survival. Also, the clearance of antioxidant components is lower in OL-HDF than in HFD. In the present study, catalase activity and TAC were significantly higher in the OL-HDF group than in the HFD group post-dialysis. Similarly, catalase activity was also higher in the OL-HDF group in the pre-dialysis state. Consequently, although the AntioxyScore decreases post-dialysis, it remains higher in OL-HDF post-dialysis, suggesting that OL-HDF might be superior to HFD in terms of maintaining antioxidant systems. Accordingly, while the DialysisOxyScore indicates that the global oxidative status in dialysis is homogeneously imbalanced in favor of oxidative stress, this is tempered by OL-HDF treatment both pre- and post-dialysis. Therefore, provided that the transition from HFD to OL-HDF accomplishes the clinical criteria [47], OL-HDF might contribute to decreased oxidative stress in the long term and thus would be preferred, at least in terms of oxidative status.

Finally, improving antioxidant systems in hemodialysis patients is of special interest due to their restrictive diet with very low amounts of fruits and vegetables [13]. Antioxidant supplementation with vitamins A, C, and E, β-carotene, or N-acetyl cysteine (NAC) reduces cardiovascular risk in hemodialysis patents [48]. In particular, patients receiving vitamin E, which prevents oxLDL formation by inhibiting lipid peroxidation [49], have a decreased rate of cardiovascular events in hemodialysis [50], suggesting that the increase in oxLDL during dialysis could be involved in the high cardiovascular risk of hemodialysis patients. Moreover, a decrease in vitamin C in hemodialysis patients is associated with major adverse cardiovascular events and cardiovascular mortality [51], although supplementation with vitamin C might lead to oxalate accumulation [52] and an increase in oxidative stress [11]. Therefore, while decreasing oxidative stress in dialysis is a promising option, the supplementation of antioxidants might not always be the most optimal alternative. Accordingly, the use of OL-HDF favors endogenous antioxidant systems without the need for external supplementation.

The major limitation of our study was the small number of included participants, and more studies are needed to confirm our results. However, the most relevant point of our study is the analysis of several parameters of oxidative stress, including oxidative damage and antioxidant defense, to compute the DialysisOxyScore. This parameter might be useful to compare results between different research groups and patient cohorts.

## 5. Conclusions

In conclusion, patients under OL-HDF treatment have a better antioxidant profile than their peers on HFD. This improvement in the endogenous antioxidant capacity might help to reduce the high cardiovascular risk of dialysis patients.

## Figures and Tables

**Table 1:** Demographic characteristics of the patients.

	All Patients (*n* = 32)	HFD Patients (*n* = 9)	OL-HDF Patients (*n* = 23)	*p*-Value
Demographic and clinical characteristics				
Age (years)	60.0 ± 16.4	64.7 ± 22.0	58.2 ± 13.8	0.3224
Sex (Men, %)	50	11.1	65.2	0.0155
BMI (kg/m2)	22.2 ± 3.9	19.6 ± 3.3	23.2 ± 3.7	0.0169
SBP (mmHg)	128.4 ± 23.0	126.2 ± 23.2	129.3 ± 23.4	0.7392
DBP (mmHg)	73.0 ± 16.7	67.8 ± 21.3	75.0 ± 14.6	0.2780
Medical history				
Hypertension (%)	78.1	77.7	78.2	0.9999
Diabetes mellitus (%)	22.8	22.2	21.8	0.9999
Current smokers (%)	12.5	0	17.4	0.3035
Dialysis time (months)	79.5 ± 74.3	31.4 ± 24.5	98.4 ± 79.1	0.0195
Laboratory measurements				
NT-proBNP (pg/mL)	6312 ± 10946	12668 ± 18811	4000 ± 5148	0.0534
25-Hydroxyvitamin D (ng/mL)	10.0 ± 4.3	9.8 ± 3.2	10.1 ± 4.7	0.8696
Total cholesterol (mg/dL)	145.8 ± 28.6	148.4 ± 25.1	144.7 ± 30.3	0.7473
Triglycerides (mg/dL)	143.3 ± 61.4	167.0 ± 52.5	134.0 ± 63.1	0.1753
Serum creatinine (mg/dL)	7.61 ± 2.22	7.16 ± 2.22	7.78 ± 2.25	0.4861
Serum albumin (g/dL)	4.05 ± 0.43	3.91 ± 0.24	4.10 ± 0.48	0.2592
CaP	38.87 ± 10.15	39.20 ± 7.27	38.74 ± 11.17	0.9095
Kt/V	1.63 ± 0.24	1.68 ± 0.25	1.61 ± 0.24	0.4628
Potassium (mEq/L)	5.15 ± 0.90	5.06 ± 0.78	5.19 ± 0.95	0.7248
Bicarbonate (mEq/L)	21.25 ± 2.94	21.22 ± 3.80	21.26 ± 2.63	0.9740
Convection volume (L/session)			29.32 ± 3.50	
Medication				
ACEi/ARB (%)	25	22.2	26.1	0.9999
Beta-blocker (%)	40.6	55.6	34.8	0.4269
Diuretics (%)	9.4	0	13.0	0.5409
Cinacalcet (%)	9.4	0	13.0	0.5409
Paracalcitol (%)	38.7	55.6	31.8	0.2534

Data from 32 patients are reported as mean ± SD or percentage. BMI: body mass index; SBP: systolic blood pressure; DBP: diastolic blood pressure; NT-proBNP: N-terminal-pro hormone B-type natriuretic peptide; ACEi: angiotensin converting enzyme inhibitor; ARB: angiotensin receptor blocker; HFD: high-flux dialysis; OL-HDF: on-line hemodiafiltration.

## Abbreviations

8-OHdG	8-hydroxy-2′-deoxyguanosine
ACEi	Angiotensin converting enzyme inhibitor
ARB	Angiotensin receptor blocker
AUC	Area under the curve
BAP	Biological antioxidant potential
BMI	Body mass index
CKD	Chronic kidney disease
DBP	Diastolic blood pressure
DM	Diabetes mellitus
ESRD	End-stage renal disease
GFR	Glomerular filtration rate
HFD	High-flux dialysis
NAC	N-acetyl cysteine
NT-proBNP	N-terminal-pro hormone B-type natriuretic peptide
OL-HDF	On-line hemodiafiltration
oxLDL	Oxidized low density lipoprotein
RRTROS	Renal replacement therapyReactive oxygen species
SBP	Systolic blood pressure
SOD	Superoxide dismutase
RRT	Renal replacement therapy
TAC	Total antioxidant capacity
XOD	Xanthine oxidase
